# Homopolymeric tracts represent a general regulatory mechanism in prokaryotes

**DOI:** 10.1186/1471-2164-11-102

**Published:** 2010-02-09

**Authors:** Renato H Orsi, Barbara M Bowen, Martin Wiedmann

**Affiliations:** 1 Department of Food Science, Cornell University, Ithaca, NY 14853, USA

## Abstract

**Background:**

While, traditionally, regulation of gene expression can be grouped into transcriptional, translational, and post-translational mechanisms, some mechanisms of rapid genetic variation can also contribute to regulation of gene expression, e.g., phase variation.

**Results:**

We show here that prokaryotes evolved to include homopolymeric tracts (HTs) within coding genes as a system that allows for efficient gene inactivation. Analyses of 81 bacterial and 18 archaeal genomes showed that poly(A) and poly(T) HTs are overrepresented in these genomes and preferentially located at the 5' end of coding genes. Location of HTs at the 5' end is not driven by a preferential placement of aminoacids encoded by the AAA and TTT codons at the N-terminal of proteins. The *inlA *gene of the pathogen *L. monocytogenes *was used as a model to further study the role of HTs in reversible gene inactivation. In a number of *L. monocytogenes *strains, *inlA *harbors a 5' poly(A) HT, which regularly shows frameshift mutation leading to expression of a truncated 8 aa InlA protein. Translational fusions of the *inlA *5' end allowed us to estimate that the frequency of variation in this HT is about 1,000 fold higher than the estimated average point mutation frequency.

**Conclusions:**

As frameshift mutations in HTs can occur at high frequencies and enable efficient gene inactivation, hypermutable HTs appear to represent a universal system for regulation of gene expression in prokaryotes. Combined with other studies indicating that HTs also enable rapid diversification of both coding and regulatory genetic sequences in eukaryotes, our data suggest that hypermutable HTs represent a general and rapid evolutionary mechanism facilitating adaptation and gene regulation across diverse organisms.

## Background

The ability to adapt to different environments is critical for all living organisms. Mechanisms of adaptation range from rapid changes in gene transcription to stable genetic changes that occur over an evolutionary time frame. Phase variation is a specific adaptive mechanism that typically involves a rapid switch between two different states (ON and OFF) to enable individual microbial cells to vary expression of proteins. Bacterial pathogens such as *Escherichia coli*, *Neisseria meningitides *and *Helicobacter pylori *are examples of organisms where phase variation, affecting the expression of flagella, fimbriae and outer membrane proteins, has been well documented [1,2]. Phase variation has also been observed in a number of non-pathogenic bacteria including *Geobacillus stearothermophilus*, *Acidithiobacillus ferroxidans*, and *Bacillus subtilis *[3].

Regulation of phase variation can occur through multiple, different mechanisms, including homologous and site-specific recombination events that generate chromosomal changes that activate or inactivate expression of a gene [1], e.g., due to inversion of a promoter element. Another effective mechanism for rapid phenotypic diversification among bacteria is through generation of genetic diversity in regions of short sequence repeats (SSRs), and, in particular in homopolymeric tracts (HTs). Frameshift mutations in HTs can occur at high frequencies and enable efficient and reversible gene inactivation [4]. The high mutation rate in HTs results from slipped-strand mispairing (SSM), which occurs during DNA replication and causes insertions or deletions (indels) [5]. Indels in HTs located in coding genes usually create a shift in the gene's translational reading frame, resulting in expression of truncated and often non-functional proteins. In particular, mutations at the 5' end of a coding gene usually lead to expression of short, non-functional peptides. For example, the *Bacillus subtilis swrA *gene, which is involved in swarming, is phase variable due to a frameshift insertion in a poly(A) HT between nt 20 and 27 [4]. In *Campylobacter coli*, a deletion in a poly(T) HT at the 5'end of *flhA *has been shown to be responsible for the phase variation of FlhA expression [6]. In the human foodborne pathogen *Listeria monocytogenes*, frameshift deletions have been observed in a 5' poly(A) HT in the virulence gene *inlA *[7]. This gene encodes the internalin protein, a virulence factor necessary for internalization into human intestinal epithelial cells and hence required for effective systemic infection [8,9]. Although the 5' frameshift mutation in *inlA *seems to be more prevalent among food isolates, a few human isolates have also been found presenting this genotype [7,10-12].

To broadly probe for the distribution of HTs in prokaryotes and to gain further insights in possible regulatory roles of HTs, the coding sequences of 99 prokaryotic (81 bacterial and 18 archaeal) genomes were scanned for the presence of HTs (for each of the four nucleotides). Our data indicate that prokaryotes have evolved to contain HTs within the 5' ends of coding sequences, probably to contribute to regulation of gene expression (e.g., phase variation, reversible gene inactivation) as frameshift mutations closer to the 5'end of coding genes would more effectively abolish expression of the full protein. Further functional studies in *L. monocytogenes *showed that the poly(A) HT located in the *inlA *5' end shows a high 6A to 7A reversion frequency, which is about 1,000 fold higher than the estimated average frequency for point mutations. Combined with other studies that also showed high mutation frequencies in other bacterial HTs, our data indicate that hypermutable HTs appear to represent a universal system for regulation of gene expression in prokaryotes.

## Results

### Poly(A) and poly(T) HTs with up to 7 bases are generally overrepresented in prokaryotic coding genes

**Figure 1:**
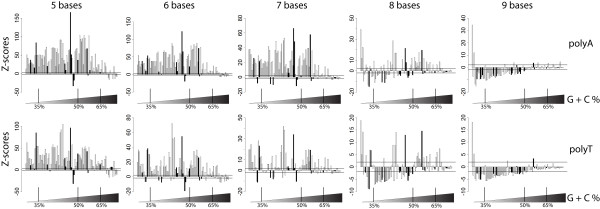
**Z-scores of the frequency of poly(A) and poly(T) HTs with 5 to 9 bases in the coding genes of 99 selected prokaryotic genomes**. Each bar represents one bacterial genome; genomes are sorted by increasing G+C content in the coding genes (a list of genomes in the order displayed here is available as Additional File 5). Bacterial genomes are represented by white bars while black bars represent archaeal genomes. Horizontal lines indicate ± 1.96 standard deviations (the cut-off for significant [*P *< 0.05] over or under-representation). Bars with Z-scores > 0 indicate an overrepresentation, while Z-scores < 0 indicate underrepresentation of the HTs in the sequences analyzed. Vertical lines at the bottom of each graph show the approximate GC content of the organisms.

Analyses of the coding genes of 99 prokaryotic (81 bacterial and 18 archaeal) genomes showed that poly(A) and poly(T) HTs ranging from 3 to 7 bases in length were significantly (*P *< 0.05; Z-test) overrepresented in the coding genes of most prokaryotic genomes evaluated (Figure 1; Additional File 1). For example, poly(A) HTs with 6 bases were overrepresented, in coding genes, of 70 of the 81 bacterial and 11 of the 18 archaeal genomes. As genomes with high GC content will have low expected frequencies of poly(A) and poly(T) HTs with 8 or more bases, genomes with high GC content do not show underrepresentation of poly(A) and poly(T) HTs (see Figure 1). For example, the expected frequency of poly(A) HTs with 8 bases for a genome with GC content of 70% and approximately 1.8 Mb of coding sequence is close to 0.3; hence it would be statistically impossible to detect an underrepresentation of poly(A) HTs with 8 bases in this genome. The converse is also true for genomes with low GC content, which will have low expected frequencies of poly(C) and poly(G) HTs with 8 or more bases. While a total of 22 genomes showed no overrepresentation of poly(A) or poly(T) HTs with 7 to 9 bases, there are no apparent common features among these genomes. Briefly, these 22 genomes represent 12 bacterial genomes and 10 archaeal genomes. The genomes include human pathogens (e.g. *Mycobacterium leprae *and *Staphylococcus aureus*) as well as fungal symbionts (i.e. *Frankia alni*) and non-pathogenic free living organisms (e.g. *Kineococcus radiotolerans*). In terms of genome features, the GC content of these 22 organisms range from 33% to 74%, the total length of coding genes range from 1,201,602 nt to 8,362,023 nt and the number of coding genes in each genome ranges from 1434 to 7769.

While poly(C) and poly(G) HTs with more than 2 bases were generally underrepresented in the genomes evaluated, poly(C) HTs with 7 and/or 8 bases were overrepresented in 11 genomes (Additional File 1) and poly(G) HTs with 7 and/or 8 bases were only overrepresented in 9 genomes (Additional File 1). Among the genomes that show overrepresentation of poly(C) or poly(G) HTs with 7 or 8 bases, only *Thermus thermophilus *has a GC content greater than 50% (%GC = 69.6).

HTs longer than 7 bases were generally not overrepresented among the coding genes evaluated, although some exceptions could be observed. For example, coding genes in 35 of 81 bacterial and 4 of 18 archaeal genomes also showed overrepresentation of poly(A) HTs with 8 bases. Overall, 41 genomes showed significant overrepresentation of poly(A) HTs with 8 and/or 9 bases, including *Buchnera aphidicola*, *Borrelia burgdorferi*, and *Chlamydia trachomatis*, three of seven organisms with less than 1000 genes in their chromosome included among the genomes analyzed here. *B. aphidicola *and *B. burgdorferi *also showed overrepresentation of poly(T) HTs with 8 and 9 bases.

Among the archaeal genomes, only the *Candidatus methanoregula *genome showed significant overrepresentation of poly(A) or poly(T) HTs with 8 and 9 bases (Additional File 1). Wilcoxon tests on Z-scores for overrepresentation of poly(A) and poly(T) HTs showed that, compared to archaeal genomes, bacterial genomes showed increased overrepresentation of poly(A) HTs with 6 to 8 bases (*P *= 0.024, *P *= 0.006, *P *= 0.009, for 6, 7 and 8 bases, respectively) and poly(T) HTs with 6 and 7 bases (*P *= 0.006 and *P *= 0.011, respectively). Furthermore, prokaryotic genomes of mammalian pathogens showed significant overrepresentation of poly(A) HTs with 6 and 7 bases (*P *= 0.007, *P *= 0.011, respectively) as compared to other prokaryotes (i.e. plant pathogens, symbionts, invertebrate pathogens and free living organisms).

As it is tempting to hypothesize that HTs may be associated with specific functional gene classes (e.g. pathogenicity factors, cell surface proteins), we also performed a preliminary analysis on the frequency of HTs among genes representing different role categories (available at [13]) in two model organisms, i.e., *L. monocytogenes *strain EGD-e and *B. aphidicola *strain Cc, which presented overrepresentation of long poly(A) and poly(T) HTs with highest deviations from the expected values among the organisms studied. These analyses did not show any clear pattern that would indicate that some specific role categories would be associated with overrepresentation of long HTs (Additional File 2). For example, only *L. monocytogenes *genes classified as "hypothetical proteins" or "regulatory functions" showed an overrepresentation of poly(T) HTs with 7 bases, while all role categories but "signal transduction" and "protein synthesis" showed overrepresentation of poly(A) HTs with 7 bases. No role categories showed overrepresentation of HTs with 8 or more bases in *L. monocytogenes*. Conversely, in *B. aphidicola*, all role categories showed overrepresentation of poly(A) HTs with 7 and 8 bases and only "regulatory functions" did not show overrepresentation of poly(A) HTs with 9 bases.

### Poly(A) and poly(T) HTs are located significantly closer to the 5'end of coding genes, suggesting a role for poly(A) and poly(T) HTs in regulation of gene expression through phase variation and reversible gene inactivation

**Figure 2:**
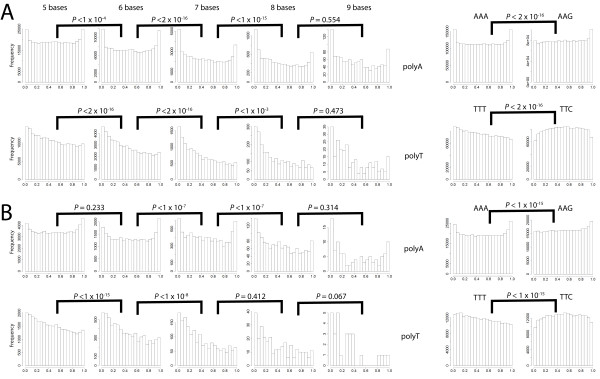
**Histograms showing the frequency of HTs in 20 windows each representing 5% of the length of the coding genes**. (i.e., the first bar represents the frequency of HTs in the first 5% of the coding genes) for **(A) **all 81 bacterial genomes and (B) all 18 archaeal genomes. The data shown represent the frequencies among all bacterial or archaeal genomes (i.e., data for all genomes were pooled and not analyzed separately for each organism). Statistical analyses indicate that longer poly(A) and poly(T) HTs are located significantly closer to the 5'end of coding genes relative to shorter tracts (*P*-values from one-sided Wilcoxon tests are shown above graphs, e.g., *P *< 1 × 10^-4 ^indicates that A_6 _tracts are located significantly closer to at the 5' ends of genes as compared to A_5 _tracts [for bacterial genomes]); AAA and TTT codons were also located significantly (*P *< 1 × 10^-15^) closer to the 5' as compared to AAG and TTC, respectively.

**Figure 3:**
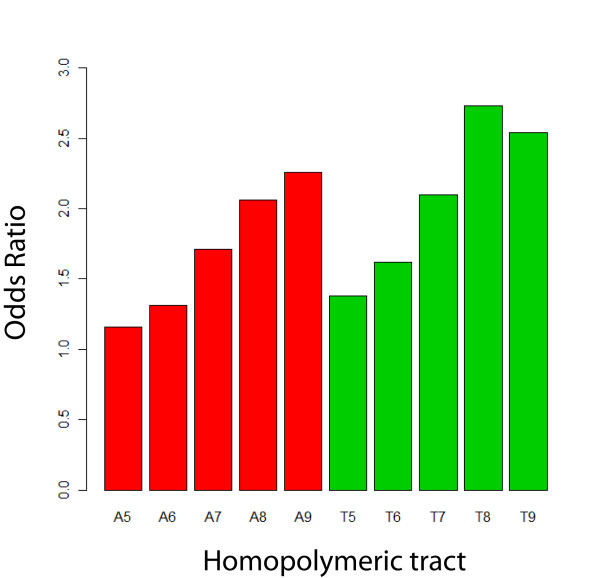
**Odds ratios of having a poly(A) or poly(T) HT with a given length in the first 10% of coding genes**. Observed frequencies for poly(A) or poly(T) HT (with length between 5 and 9 nt) located in the region encompassing the 5' 10% of all genes (based on pooled data for all 99 genomes) were compared to expected frequencies (calculated based on a randomly generated dataset created under the assumption that the location of the respective HTs are uniformly distributed within coding genes). Y axis shows the odds ratios, e.g. an odds ratio of 2.3 for an A_9 _tract indicates that A_9 _HTs are 2.3 fold more likely to occur in the first 10% of a coding gene as compared to their expected frequency. The lower limits of 95% confidence intervals for all Odds Ratios were > 1, indicating that all poly(A) or poly(T) HT shown (i.e, those with sizes between 5 and 9 nt) occur significantly more commonly in the 5' 10% of genes in the prokaryotic genomes evaluated than expected by chance.

We hypothesized that if HTs evolved to contribute to regulation of gene expression (e.g., phase variation, reversible gene inactivation), selective pressures would favor the placement of the HTs near the 5' end of the reading frame, where frameshift mutations would more effectively abolish expression of the full protein. Therefore, the relative position of HTs (ranging from 5 - 10 bases in length) within a given coding gene was also investigated among the 81 bacterial and 18 archaeal genomes analyzed. As the number of bases in poly(A) and poly(T) HTs increased from 5 to 8, the HTs were located closer to the 5' end of coding genes (Figure 2). However, HTs > 8 bases as well as poly(C) and poly(G) HTs were not located significantly closer to the 5'ends of coding genes. To further assess whether the relative locations of poly(A) and poly(T) HTs with 5 to 8 bases were significantly biased towards the 5'end of coding genes, we compared the observed number of a HT in the first 10% of coding genes (using pooled data from all genomes) against the expected number of the same HT (in the same region) under the assumption that HTs are uniformly distributed within a given gene. The observed numbers of HTs in the first 10% of coding genes was significantly greater for all HTs as compared to the expected number of HTs (*P *< 0.001; Fisher's exact test). Moreover, the odds ratio of having an HT in the first 10% bases of coding genes in the observed dataset as compared to the expected dataset generally increases as the number of bases in the HT increases (Figure 3).

Overall, poly(A) HTs with 7 and 8 bases were located significantly closer to the 5'end of the genes in the genomes of mammalian prokaryotic pathogens as compared to other prokaryotes (i.e. plant pathogens, symbionts, invertebrate pathogens and free living organisms) (*P *< 0.001 and *P *= 0.047, respectively; Wilcoxon one-sided test). Poly(T) HTs, on the other hand, were not located closer to the 5' end in the genes of mammalian prokaryotic pathogens as compared to other prokaryotes (*P *> 0.05; one-sided Wilcoxon test). Wilcoxon one-sided tests also revealed that, compared to archaeal genomes, poly(A) HTs with 5 to 7 bases from bacterial genomes are located significantly closer to the 5'end of coding genes (*P *< 0.001 for all HTs). Conversely, compared to bacterial genomes, poly(T) HTs with 6 bases from archaeal genomes are located significantly closer to the 5' end of genes (*P *= 0.020).

### While AAA and TTT codons are preferentially located at the 5' end of genes, the same is not observed for AAG and TTC codons, which encode the same amino acids as AAA and TTT

To test whether the positioning of poly(A) and poly(T) HTs was associated with the placement, at the N-terminal end of proteins, of amino acids encoded by the AAA or TTT codons, we analyzed the distribution of these two codons as well as the distribution of the AAG and TTC codons, as these pairs of codons encode the same two amino acids, i.e., lysine (Lys, encoded by AAA and AAG) and phenylalanine (Phe, encoded by TTT and TTC). Analyses of the coding sequences from all 99 genomes tested here found that both AAA and TTT codons are located significantly closer to the 5' end of the coding genes as compared to the AAG and TTC codons, respectively (*P *< 1 × 10^-15 ^for both, Wilcoxon one-sided test; see Figure 2). These results suggest that the location of poly(A) and poly(T) HTs closer to the 5'end of coding genes is not a consequence of a preferential location of the Lys and Phe amino acids at the N-terminal of prokaryotic proteins.

When compared to a randomly generated uniformly distributed dataset of pseudo relative locations, Lys was located significantly closer to the C-terminal ends of proteins (*P *< 1 × 10^-15^, Wilcoxon one-sided test) than it would be expected if the relative locations of Lys were uniformly distributed. The C-terminal enrichment of Lys can not be explained by the distribution of poly(A) HTs as 39% of the Lys at the C-terminal are encoded by the AAG codon and poly(A) HTs are located significantly closer to the 5'end of genes. We thus investigated the location of all other 19 amino acids in all coding genes of the 99 prokaryotic genomes and observed that, in additions to Lys, Arginine (Arg) was also located in the C-terminal of proteins. This C-terminal placement of Arg was also statistically significant (*P *< 1 × 10^-15^, Wilcoxon one-sided test using a comparison of the observed data to a randomly generated uniformly distributed dataset of pseudo relative locations). As Lys and Arg are similar amino acids (i.e., both are basic, large and acyclic) these data suggest that the C-terminal enrichment for Lys is associated with the biochemical characteristics of the amino acid itself.

### Position of poly(A) and poly(T) HTs at 5' ends of genes is not associated with AAA and TTT codon usage

One possible explanation for a bias towards AAA and TTT codons at the 5' end of genes could be selection for common (preferred) codons at 5' end of genes to improve translational efficiency and avoid ribosome stalling. We thus assessed whether the position of poly(A) and poly(T) HTs was associated with codon usage patterns for AAA and TTT codons. Among the 99 prokaryotic genomes analyzed, AAA and TTT were preferred codons (usage >50%) for 61 and 63 genomes, respectively. On the other hand, for 26 and 29 genomes, AAG and TTC codons represented the preferred codons; in these genomes AAG and TTC represented > 70% of the Lys and Phe codons, respectively. Even among these genomes with high preference for codons AAG and TTC, significant clustering of AAA and TTT codons in the 5'end of genes was observed (*P *< 1 × 10^-15 ^for both, Wilcoxon one-sided test). Overall, these data suggest no association between the positioning bias of HTs and codon bias and thus indicate that the position of poly(A) and poly(T) HTs within prokaryotic coding genes is not driven by codon bias.

### A 5' poly(A) HT in *L. monocytogenes inlA *facilitates high frequency adenosine insertion that restores *inlA *translational frame

To further investigate the role of HTs in phase variation and gene inactivation, we used the facultative intracellular pathogen *L. monocytogenes *as a model. *L. monocytogenes *shows significant positioning of poly(A) HTs with up to 7 bases closer to the 5'end of genes; A_7 _HTs occur with a frequency of approx. 0.45 HTs per coding gene (Additional File 1). A_7 _and T_7 _tracts are also located significantly closer to the 5'end of genes (as compared to A_6 _and T_6 _tracts, respectively; *P *< 0.001 and *P *= 0.002, Wilcoxon one-sided test) in the *L. monocytogenes *genome.

We specifically used a *L. monocytogenes *poly(A) HT in the 5' end of *inlA *as a model to investigate the mutation frequency of poly(A) HTs. The 5' *inlA *poly(A) HT was previously shown to be polymorphic in *L. monocytogenes*. In one phylogenetic *L. monocytogenes *lineage (lineage II) the 5' end of *inlA *either includes an A_7 _HT, which leads to an in-frame *inlA*, or an A_6 _HT, which causes a frameshift mutation with a premature stop codon after aa 8 [7]. Interestingly, all *L. monocytogenes *isolates in lineage I characterized so far as well as some lineage II isolates carry a "AAGAAAA" (A_2_GA_4_) sequence (which leads to an in-frame *inlA*) instead of an A_7 _or A_6 _HT [7]. Translational out of frame fusions between the three different allelic variants at the 5'end of *inlA *and *kanR*, a kanamycin resistance gene, were created in *L. monocytogenes *FSL F2-640 (Table 1) to investigate the frequency of frameshift mutations including the frequency of (i) A_6 _→ A_7 _and (ii) A_7 _→ A_6_, and (iii) the frequency of deletions in A_2_GA_4_; in each case, the mutation of interest would revert an out-of frame *kanR *fusion to an in-frame *kanR *fusion that yields a kanamycin resistance phenotype. In three biological replicates, the strain carrying an A_6 _out of frame *kanR *fusion showed between 4,600 and 35,000 revertants per 2 to 3 × 10^10 ^bacteria, indicating an A_6 _→ A_7 _reversion frequency of approx. 5.5 × 10^-7 ^(based on an average of three experiments; see Additional File 3). On the other hand, only a single revertant was obtained in one of the three replicate experiments with the strain carrying an A_7 _out of frame *kanR *fusion, indicating a low A_7 _→ A_6 _reversion frequency (which can be estimated to be approx. 3.9 × 10^-8^; see Additional File 3). In three biological replicates, no kanamycin resistant revertants with a deletion in the A_2_GA_4 _region were observed, indicating a very low frequency of deletions (below approx. 3 × 10^-10^; see Additional File 3 for detailed data).

**Table 1:** Strains used for assessment of frameshift frequencies

Strain	Characteristics
FSL F2-640	Parent strain, used for amplification of the A_6 _*inlA *5' allele for generation of *kanR *fusion strains
EGD-e	Parent strain for construction of *kanR *fusion strains
FSL B2-112	In-frame A_6 _*inlA *5' allele -*kanR *fusion (control strain)
FSL B2-122	Out of-frame A_6 _*inlA *5' allele -*kanR *fusion; used to assess frequency of A_6 _→ A_7 _insertion
FSL B2-135	Out of-frame A_7 _*inlA *5' allele -*kanR *fusion; used to assess frequency of A_7 _→ A_6 _deletion
FSL B2-136	Out of-frame A_2_GA_4_*inlA *5' allele -*kanR *fusion; used to assess frequency of deletions in A_2_GA_4_

### The frameshift mutation in a poly(A) HT in the 5' end of *L. monocytogenes inlA *has at least two independent origins

**Figure 4:**
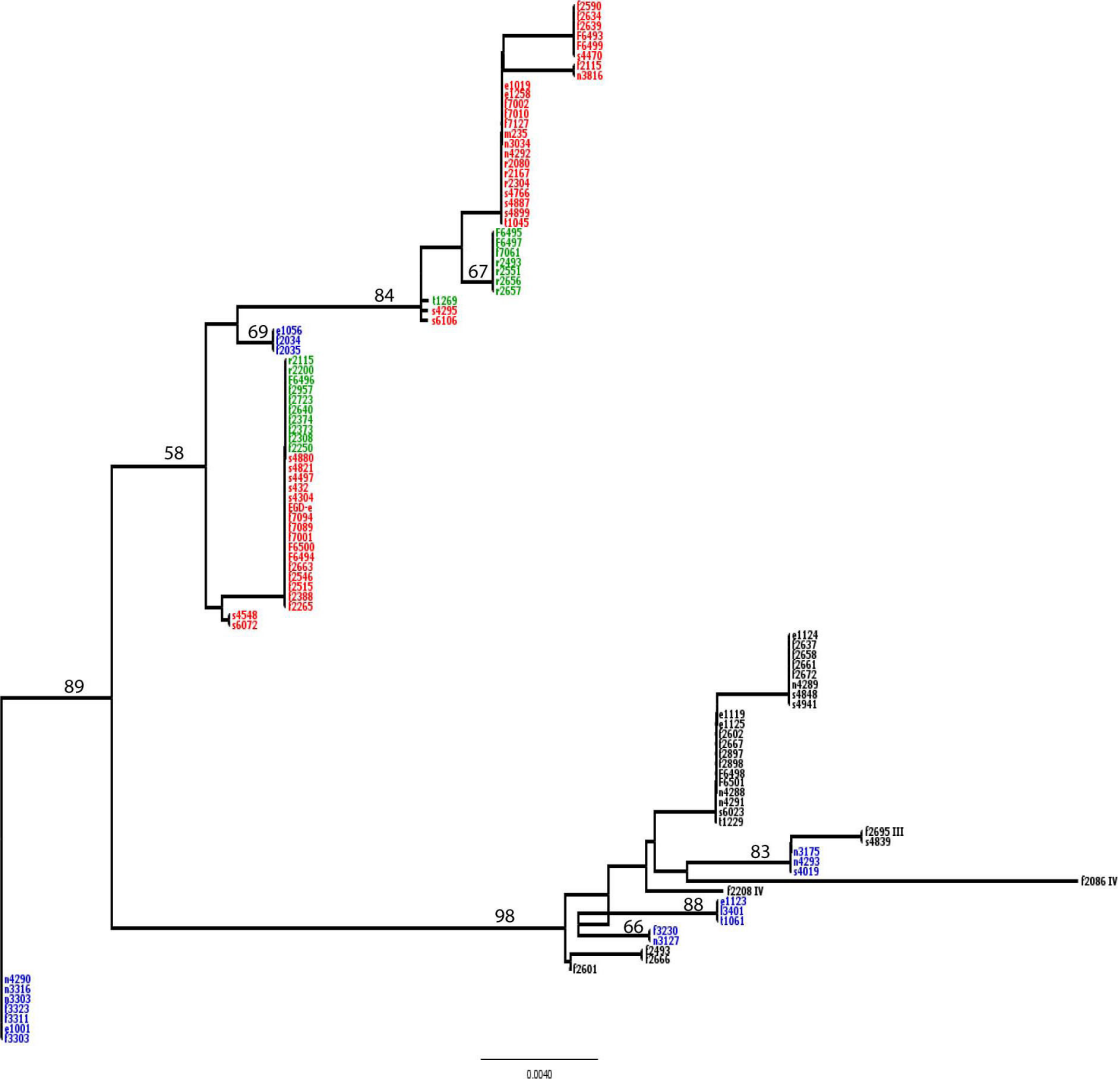
**Neighbor-Joining tree of an 420 nt sequence that includes the 5' end fragment of *inlA *(194 nt) and the upstream region (226 nt)**. Clades representing lineages I, II, III, and IV are indicated. *inlA *sequences for *L. monocytogenes *lineage II isolates are color-coded; isolates bearing the A_6 _allele are shown in green; isolates bearing the A_7 _allele are shown in red; and isolates bearing the A_2_GA_4 _allele are shown in blue. Lineage I, III (F2695) and IV (F2086 and F2208) isolates are shown in black and they all bear the A_2_GA_4 _allele. The tree is unrooted. Bootstrap values are shown on top of their respective selected branches.

To further investigate the origin of the frameshift mutation in *L. monocytogenes inlA*, the DNA sequences including the noncoding region upstream *inlA *as well as the 5' coding region of the gene from 106 *L. monocytogenes *isolates (Additional File 4) were used to probe the phylogenetic history of this fragment in these isolates. The frameshift mutation seems to have arisen at least twice, independently, among *L. monocytogenes *lineage II isolates (Figure 4). Interestingly, one of the clusters containing *inlA *sequences with the A_6 _allele (which results in the premature stop codon) also contained isolates with the A_7 _allele. In this clade, the only difference between the *inlA *sequences with A_6 _HT and the *inlA *sequences with the A_7 _HT was the single adenine indel in this HT.

Phylogenetic analyses of *inlA *sequences also showed that all *inlA *sequences for *L. monocytogenes *lineage I isolates (which predominantly represent serotypes 1/2b and 4b) have an A_2_GA_4 _instead of an A_7 _(or A_6_) sequence. Among the 79 lineage II isolates only 18 carried this A_2_GA_4_*; *the other lineage II isolates carried either an A_7 _(43 isolates) or an A_6 _(18 isolates) sequence at this location. The 18 lineage II *inlA *sequences with an A_2_GA_4 _sequence at the 5' end formed five cluster, including three clusters that were located within the clade that contained all lineage I *inlA *sequences, suggesting that these lineage II isolates acquired this allele from a lineage I ancestor through horizontal gene transfer followed by homologous recombination. The observation that the other *inlA *sequences with an A_2_GA_4 _sequence at the 5' end of *inlA *formed two distinct clusters within the clade that contained only lineage II *inlA *sequences, suggests that these two clusters with an A_2_GA_4 _sequence 5' end of *inlA *arose at least twice independently, suggesting a strong selective pressure for this change.

## Discussion

Genome wide analyses on 99 prokaryotic genomes (including 81 bacterial and 18 archaeal genomes) as well as in-depth characterization of evolutionary history and reversion frequency in a poly(A) homopolymeric tract in *L. monocytogenes inlA *were performed to evaluate the role of homopolymeric tracts as general regulatory features allowing for rapid gene silencing and phase switching. Overall, our data indicate that (i) poly(A) and poly(T) homopolymeric tracts are overrepresented in prokaryotic genes and preferentially located at the 5' ends of genes, indicating selective pressure for presence of these homopolymeric tracts consistent with their role as regulatory elements, and (ii) poly(A) homopolymeric tracts show high mutation frequency in *L. monocytogenes *and are used to selectively inactivate the virulence gene *inlA*.

### Poly(A) and poly(T) homopolymeric tracts are overrepresented in prokaryotic genes and preferentially located at the 5' ends of genes

Our analysis of homopolymeric tracts in coding genes of 99 prokaryotic genomes showed that poly(A) and poly(T) HTs with 3 to 7 bases are overrepresented in most of these genomes while poly(C) and poly(G) tracts are not. Interestingly, mammalian pathogens showed increased overrepresentation of poly(A) HTs with 6 and 7 bases as compared to other prokaryotes (i.e., plant pathogens, symbionts, invertebrate pathogens and free living organisms). This may reflect the higher need for phase-variable antigens among mammalian pathogens in order to evade the host immune system [1]. For example, several *H. pylori *outer membrane proteins were shown to be phase variable during murine infection [14]. Additional putative targets of the mammalian immune system were shown to be phase variable in other pathogens, including *Mycoplasma *spp [15], *Burkholderia mallei *[16], and *Bordetella pertussis *[17], among others. The only organism, among those analyzed, with overrepresentation of poly(A) and poly(T) tracts with 8 to 10 nt was the endosymbiont *B. aphidicola*. Poly(A) HTs had previously been shown to be overrepresented in the *B. aphidicola *genome, which is extremely AT rich [18]. While frameshift mutations were observed in several poly(A) tracts in *B. aphidicola*, transcriptional polymerase slippage appears to generate heterogeneous mRNAs for these genes, including a number of mRNAs encoding in-frame proteins, suggesting that at least in some organisms frameshifts in homopolymeric tracts may not always fully inactivate gene function.

Overall, our data also showed that longer poly(A) and poly(T) HTs are located significantly closer to the 5' ends of genes as compared to shorter tracts in prokaryotic genomes. As there was no association between the location of these HTs close to the 5' end of coding genes and either (i) codon bias or (ii) placement of amino acids encoded by AAA and TTT codons (i.e., Lys and Phe), these data support the hypothesis that the location of HTs at the 5' end of coding genes was driven by selection for DNA sequences that provide an effective mechanism for high frequency mutations, which would permit phase variation or reversible gene inactivation. As the frequency of frameshift mutations tends to increase as the HT length increases [19,20], longer HTs located closer to the beginning of genes are likely to allow for more efficient phase variation and gene inactivation as compared to shorter HTs.

Our hypothesis that homopolymeric tracts located at the 5' ends of prokaryotic coding genes provide a general mechanism for effective phase variation or reversible gene inactivation in the majority of prokaryotes is also supported by a number of previous studies on specific organisms or specific genes. For example, in *B. subtilis*, frameshift mutations in a poly(A) HT at the 5'end of *swrA *occur at high frequency, roughly 10^-4 ^and 10^-5 ^for a base pair deletion (9 to 8 bp) and insertion (8 to 9 bp), respectively [4]. A *C. coli *poly(T) HT at the 5'end of *flhA *showed a deletion frequency of 3 × 10^-4 ^(T_8 _→ T_7_) and an insertion frequency of 7 × 10^-6 ^(T_7 _→ T_8_) [6]. In *Mycoplasma hominis*, a 5'poly(A) HT in *vaa*-2 is associated with phase variation of the Vaa antigen. The switching frequency between the Vaa^+ ^(HT with 8 bp) and Vaa^- ^(HT with 7 bp) phenotypes was estimated as 10^-3 ^to < 10^-4 ^[21]. In our model, the *L. monocytogenes inlA *5' HT, we observed a frequency of insertion of an adenine in the A_6 _tract (A_6 _→ A_7_) in the order of 10^-7^, while the frequency of an adenine deletion in this HT (A_7 _->A_6_) was lower, suggesting that mutation events that lead to reversion to a full length *inlA *are more common in this HT. Further examples of HTs involved in phase variation include a poly(A) HT at the 5'end of the *Mycoplasma fermentans p78 *gene, which encodes the ABC transporter subunit p78 [15]. In this HT, deletion of one adenine in the 7-base tract was shown to result in inactivation of the gene. Frameshift mutations in a 5' poly(G) HT in *flhB *from *Pseudomonas putida *have also been described [22]. Interestingly, our data showed that *H. pylori *is the only species in our dataset with overrepresentation of poly(A), poly(C), poly(G), and poly(T) HTs with 3 to 8 bases. Consistent with this observation, there are many examples of *H. pylori *genes that are phase variable due to indels in HTs [14,23-25]. For example, Tannaes et al. (2001) reported a poly(G) HT involved in phase variation of *H. pylori pldA*, which encodes the phospholipase A. This gene has been shown to be phase variable during murine colonization of the gastric environment by *H. pylori *[14]; gene inactivation occurs by insertion of a guanine in a 7 nt poly(G) HT in *pldA*.

### Poly(A) homopolymeric tracts show high mutation frequency in *L. monocytogenes *and are used to selectively inactivate the virulence gene *inlA*

As initial data have shown that a 5' poly(A) HT in *inlA*, which encodes the *L. monocytogenes *internalin A protein, shows frequent frameshift mutations that result in a truncated and inactive 8 aa peptide [7,10], we used this gene as a model to further probe the contributions of HTs to phase-shifting and reversible gene inactivation in bacterial pathogens. Natural *L. monocytogenes *isolates have been shown to either carry an A_7_, A_6_, or A_2_GA_4 _sequence starting at *inlA *nt 6; while an A_7 _or an A_2_GA_4 _sequence at this location maintain an in-frame *inlA *ORF, an A_6 _sequence at this location leads to the truncated 8 aa peptide. Although *L. monocytogenes *represents at least four phylogenetic lineages (i.e., lineages I, II, III and IV) [26-28], A_7 _or A_6 _HTs have so far only been identified in the *inlA *gene of lineage II isolates (even though some lineage II strains harbor *inlA *sequences with an A_2_GA_4 _at the 5' end). All *inlA *sequences for isolates representing other lineages (I, III, and IV) were found to harbor an A_2_GA_4 _sequence at the 5' location of *inlA*, which our data indicate represents a stabilized HT that shows a lower mutation frequency as compared to the poly(A) HT at this location. The frequency of an A_6 _→ A_7 _reversion was estimated as approx. 1,000 fold higher than the estimated average point mutation frequency (i.e., 4.5 × 10^-10 ^[29]). Although the experimentally observed reversion frequency from A_7 _→ A_6 _is not significantly different from the lack of any revertants observed for A_2_GA_4_, our population genetics studies clearly show that the deletion A_7 _→ A_6 _occurred at least twice, indicating that this mutation occurs in nature and is biologically relevant. Interestingly, while *L. monocytogenes *lineage I strains are overrepresented among human disease associated isolates and lineage III isolates are overrepresented among animal disease associated isolates [30,31], lineage II strains are overrepresented among foods and non-clinical disease associated isolates, even though they are regularly isolated from human and animal listeriosis cases [30,31]. Hence it appears that disease associated *L. monocytogenes *have evolved an *inlA *sequence that facilitates stable expression of this virulence gene, which is essential for infection [8,32,33], while some generalist strains (i.e., some lineage II strains) have evolved mechanisms that allow for reversible silencing of *inlA*, possibly during environmental survival or inside hosts where *inlA *is not required (e.g., mice [34]). Interestingly, some lineage II strains have acquired, through multiple independent events, an A_2_GA_4 _sequence at the *inlA *5' end, possibly suggesting adaptation, of some clades and strains within lineage II, to a more disease associated lifestyle (consistent with a previous data that identified human and animal disease associated clades in lineage II [35]). These data suggest that evolutionary forces select for presence of HTs in specific prokaryotic genes and genetic lineages, possibly to facilitate adaptation to specific ecological niches and lifestyles (e.g., host adapted life styles). As our genome wide analyses suggest broad selection for presence of HTs and their location in the 5' region of coding genes in prokaryotic genomes in general, HTs thus appear to play important roles in facilitating adaptation and gene regulation in prokaryotes. Future phenotypic and genetic studies will be needed though, to clarify the biological relevance of HTs in *L. monocytogenes *and other prokaryotes. For example, even though *L. monocytogenes *showed a considerable number of genes with poly(A) and poly(T) HTs, beyond our data reported here, no functional analyses of long HTs in *L. monocytogenes *have been reported. In addition, none of the previous genome analyses in *Listeria *have included a comprehensive analysis of frameshift mutations, even though one HTs in *L. monocytogenes flaR *has been found to show evidence for phase variation based on population data [36].

## Conclusions

Based on our findings, we propose that HTs, particularly those at the 5' end of coding genes, represent a universal prokaryotic system that allows for effective gene inactivation and phase switching. This system for gene regulation is considerably slower than rapid transcriptional and translational mechanism of gene regulation, but faster and more reversible than other chromosomal mutational events. As an emerging body of evidence also supports that HTs also enable rapid diversification of both coding and regulatory genetic sequences in eukaryotes [37,38], HTs appear to represent a universal mechanism for mutational adaptation across diverse life forms.

## Methods

### Homopolymeric tract search

The coding genes from 81 bacterial and 18 archaeal completed genomes were retrieved from NCBI (Additional file 5). The 99 genomes were selected to represent the phylogenetic and ecologic diversity of prokaryotic organisms among genomes sequenced to date. We specifically selected the genomes studied here using the following criteria: (i) availability of a finished genome; (ii) inclusion of widely studied pathogens (e.g., *Salmonella*, *Bacillus anthracis*) and other widely studied organisms (e.g., endosymbionts) (in order to allow others studying these organisms to use our data); (iii) inclusion of only one representative per species (with exception of two *E. coli *and two *L. monocytogenes *genomes); (iv) representation of different groups of bacteria (i.e., Actinobacteridae, Alpha-proteobacteria, Aquificales, Archaeoglobi, Bacillales, Beta-proteobacteria, Chlamydiales, Chroococcales, Clostridia, Dehalococcoidetes, Deinococci, Delta-proteobacteria, Epsilon-proteobacteria, Gamma-proteobacteria, Halobacteria, Lactobacillales, Methanococci, Methanomicrobia, Mollicutes, Prochlorales, Spirochaetales, Thermococci, Thermoplasmata, Thermoprotei, and Thermotogales); (v) representation of a wide range of GC content (21% to 74%) and genome sizes (357 to 7769 coding genes). A list of the organisms used is provided in Additional File 1. All files were scanned for the presence of single nucleotide repeats ranging from 1 to 20 bases in length using the program "dreg" implemented in the EMBOSS package [39]. HTs with 1 to 5 bases were analyzed to allow for comparison to the longer and meaningful HTs with 6 or more bases. It is unlikely that HTs with less than 6 bases would allow for frequent insertions or deletions. To our knowledge, the shortest HTs that have been found to be involved in phase switching or a similar variation is 6 bases long (Stibitz et al., 1989; Gogol et al 2007; this work). HTs identified by dreg do not overlap (i.e. HTs with 7 bases are not counted within HTs with 8 bases). Codons were scanned using the program "fuzznuc" also implemented in the EMBOSS package.

### Statistics used to evaluate overrepresentation of HTs in the coding genes of a given genome

To estimate the expected number of HTs for each chromosome the following formula was applied:

Where:

*i *∈ {A, C, T, G}

*k *∈ {1, 2, ..., 20}; *k *is the number of bases in the homopolymeric tract

*f*_*i *_= frequency of nucleotide *i *in the coding genes

*N *= total length of the coding genes

*n *= total number of coding genes

The significance of the difference between the observed and expected values was assessed as the probability of observing a value as extreme as that observed under the null hypothesis that the number of homopolymeric tracts follows a binomial distribution. A binomial approximation to the normal distribution was used to calculate the Z-score where:

A Z-score > 1.64 (*P *< 0.05, one-sided test) was considered significant when testing for overrepresentation of a given HT.

### Statistics used to evaluate location of HTs in different gene regions

To assess clustering of HTs in different gene regions, data on the relative location, within a given gene, of HTs with a given length (e.g., T_7_) were pooled, separately, for (i) all 81 bacterial genomes and (ii) all 18 archaeal genomes analyzed. Relative location of an HT in a gene was defined as the absolute location (i.e., location of the first nt of the HT + the location of the last nt in the HT/2) divided by the length of the gene. One-sided Wilcoxon rank sum tests were performed using R (version 2.5.0) to determine whether HTs with a given length (e.g., T_6_) had relative locations significantly lower (i.e. the HTs located significantly closer to the 5' end of coding genes) as compared to the relative locations of HTs that were one base shorter (e.g., T_5_). The same general approach was used to investigate the position of AAA, AAG, TTT, and TTC codons in different gene regions. In addition, we evaluated the location of HTs in the 5'end of coding genes in different groups of organisms (i.e. mammalian pathogens, bacterial organisms, archaeal organism) by pooling the HT relative locations of all members of a group and comparing it, using the Wilcoxon rank sum one-sided test, against the relative locations for the same HT in another group.

To assess if a given amino acid was located significantly closer to the C- or N-terminal of proteins, the relative location of each amino acid was pooled among all 99 genomes. A random dataset of uniformly distributed pseudo relative locations was generated for each amino acid using the "runif" function in R. The randomly generated datasets had the same length as their respective observed dataset. The observed data was then compared to the randomly generated dataset using the Wilcoxon rank sum one-sided test.

Odds ratios and Fisher's exact tests were used to assess whether the observed numbers of poly(A) and poly(T) HTs located in the regions encompassing the 5' 10% of coding genes were significantly greater than expected under the assumption that HTs are uniformly distributed across the coding genes. A random dataset of uniformly distributed pseudo relative locations was generated for each poly(A) or poly(T) HTs with 5 to 8 bases using the "runif" function in R. The randomly generated datasets had the same length as their respective observed dataset. The number of HTs falling in the first 10% of the coding genes were then determined for the observed and expected (randomly generated) datasets and Fisher's exact test was used to compare observed and expected counts.

### *inlA-kanR *translational fusion construction

In order to evaluate the frequency of different frameshift mutations in a poly(A) HT in *L. monocytogenes*, three different alleles of the promoter region and 5' end of *inlA *gene were fused out-of-frame to the kanamycin resistance gene (*kanR*) using SOEing PCR. The *kanR *gene was amplified from plasmid pDG780 [40] and SOEing PCR was carried out as previously described [41,42]. Constructs were introduced into the *inlA *locus of EGD-e by homologous recombination as previously described [43]. The three alleles used for this fusions contained different poly(A) HT sequences in the 5' of *inlA *(starting at nt 6), including (i) an A_7 _HT, (ii) an A_6 _HT, and (iii) an A_2_GA_4 _sequence (see Table 1). In these three fusions, an (i) A_6 _→ A_7 _insertion, (ii) an A_7 _→ A_6 _deletion, and (iii) a single nucleotide deletion in A_2_GA_4 _lead to an in-frame *kanR *construct. As a control, we also constructed a *L. monocytogenes *strain that contained an in-frame *kanR *fused to the 5' end of a *inlA *genes with an A_6 _HT (see Table 1).

### Detection of mutation frequencies in *inlA *HTs using the *inlA-kanR *translational fusion strains

*L. monocytogenes kanR *fusion strains (see Table 1) were initially grown overnight in 5 ml of BHI broth without antibiotic at 37°C. Subsequently, 50 μl of the overnight cultures were inoculated into 5 ml of fresh BHI broth and incubated at 37°C with agitation until cultures reached mid-log phase (defined as on OD_600 _of approx. 0.4); 50 μl of this log-phase culture were inoculated into 5 ml of fresh BHI broth and incubated at 37°C with agitation. After incubation for 18 hours, appropriate dilutions of each culture were plated on BHI without antibiotics (to estimate the total number of viable cells) and BHI containing 150 μg/ml of kanamycin (to enumerate *kanR *revertants). Total viable counts were enumerated after 24 h of incubation at 37°C. Kanamycin resistant bacteria were enumerated using digital images of the plates, which were acquired with a Q-Counter (Spiral Biotech, Norwood MA)) after 28 and 48 h of incubation. Colonies observed on these plates clearly represented two distinct sizes, i.e., large and small colonies. The digital images were thus used for enumeration of colonies, including classification of colony size (in pixels [px]). Colonies with > 8 px were classified as "large colonies" while colonies < 9 px were classified as "small". This classification was necessary as it was observed that small colonies represented in their great majority slow growing non-revertants (possibly due to other mutations) while large colonies represented mostly true revertants. To calculate the reversion frequency for each fusion, the *inlA *fragment upstream of the *kanR *gene was sequenced for all or a proportion of large colonies; if < 20 large colonies were isolates in a given replicate for a give fusion, all large colonies were tested, otherwise at least 10 colonies were tested.

### Calculation of frameshift frequencies

The original cell density (OCD) was calculated as:

Where:

CFU = average number of colonies per plain BHI plate

VP = Volume plated in ml

DF = Dilution factor

The revertants density (RD) was calculated as:

Where:

VP = Volume plated in ml

CS = Number of large colonies sequenced from plates

R = Number of revertants confirmed by DNA sequencing

TC = Total number of large colonies

DF = Dilution factor

The frequency of revertants (FR) was calculated as:

### Phylogenetic analysis of the 5'end *inlA *fragment and upstream region

An alignment of a 420 nt fragment of the 5'end of *inlA *(194 nt) and an upstream region (226 nt) for 106 *L. monocytogenes *isolates (see Additional File 4) was used to construct a Neighbor-Joining tree in PAUP [44] using the HKY85 distance. The inlA-proF and inlA-proR primers [7] were used to amplify and sequence these fragments. The *inlA *alignment was deposited in GenBank under accession numbers GQ452120-GQ452224.

## Authors' contributions

RHO outlined, performed and interpreted the computational analyses, outlined and interpreted experimental analyses using translational fusion and drafted the manuscript. BMB constructed translational fusions of *inlA *and mutant strain of *L. monocytogenes*, and performed the experiments for frameshift frequency in *inlA*. MW supervised the project, participated in the design of the study and data interpretation, and finalized the manuscript. All authors read and approved the final manuscript.

## Supplementary Material

Additional file 1 **Distribution of homopolymeric tracts in coding genes of 99 prokaryotic chromosomes**. Columns contain species name, NCBI accession numbers of their respective chromosomes, nucleotide frequencies, GC content, total length of the coding genes summed in each chromosome, total number of coding genes in each chromosome, observed and expected numbers and an associated Z-score for each HT in each chromosome. Click here for file

Additional file 2 **Distribution of homopolymeric tracts in *Listeria monocytogenes *strain EGD-e and *Buchnera aphidicola *strain Cc coding genes classified by role categories**. Columns contain role categories for *L. monocytogenes *and *B. aphidicola*, nucleotide frequencies, GC content, total length of the coding genes summed in each role category, total number of coding genes in role category, observed and expected numbers and an associated Z-score for each HT in each role category. Click here for file

Additional file 3 **Frequency of indels in the *L. monocytogenes inlA *5'polyA HT (as determined by translational kanamycin resistance reporter fusions)**. Summary of the translational kanamycin resistance reporter fusion results for three strains representing three different alleles of the 5'end HT found in *L. monocytogenes inlA*. Three replicates were carried out for each strain. Click here for file

Additional file 4 **Isolates used for sequence analysis of the *inlA *poly(A) HT**. The table provides (i) isolate identification number, (ii) source, (iii) year of isolation, (iv) state and country of isolation, (v) lineage (I, II, III or IV), and (vi) EcoRI ribotype designations for the 105 *L. monocytogenes *isolates that were used to build a phylogeny of the 5'end of *inlA *(shown in Figure 4). Click here for file

Additional file 5 **Genomes sorted by G+C content**. Genomes used for HT analyses sorted from the lowest to highest G+C content. This is the order used in Figure 1. Click here for file
