# Supplementary material for: Homopolymeric tracts represent a general regulatory mechanism in prokaryotes
Source: BMC Genomics. 2010 Feb 9;11:102. doi: 10.1186/1471-2164-11-102 (PMC2831843; doi:10.1186/1471-2164-11-102)
Supplement: Additional file 3 — Frequency of indels in the L. monocytogenes inlA 5'polyA HT (as determined by translational kanamycin resistance reporter fusions). Summary of the translational kanamycin resistance reporter fusion results for three strains representing three different alleles of the 5'end HT found in L. monocytogenes inlA. Three replicates were carried out for each strain. [file 1471-2164-11-102-S3.DOC]

**Additional File 3.** Frequency of indels in the *L. monocytogenes inlA* 5’polyA HT (as determined by translational kanamycin resistance reporter fusions)

| Strain | | Replicate | | Original cell density (per ml) | No. of kanR colonies detected (dilution factor)^a^ | No. of revertants/no of kanR colonies tested^b^ | Calulated no. of revertants per ml^c^ | Proportion of revertants |
| --- | --- | --- | --- | --- | --- | --- | --- | --- |
| FSL B2-122 | | |  |  |  |  |  |  |
|  | 1 | | | 2.8 × 10^10^ | 8 (2 x 10^-4^) | 7/8 | 3.5 × 10^4^ | 1.3 × 10^-6^ |
|  | 2 | | | 2.2 × 10^10^ | 16 (2 x 10^-3^) | 11/16 | 5.5 × 10^3^ | 2.5 × 10^-7^ |
|  | 3 | | | 2.8 × 10^10^ | 23 (2 x 10^-3^) | 4/10 | 4.6 × 10^3^ | 1.6 × 10^-7^ |
| FSL B2-135 | | |  |  |  |  |  |  |
|  | 1 | | | 2.4 × 10^10^ | 7 (2 x 10^-3^) | 0/7 | < 5.0 × 10^2^ | < 2.1 × 10^-8^ |
|  | 2 | | | 2.6 × 10^10^ | 9 (1 x 10^-2^) | 1/9 | 1.0 × 10^2^ | 3.9 × 10^-8^ |
|  | 3 | | | 2.4 × 10^10^ | 41 (1 x 10^-2^) | 0/20 | < 1.0 × 10^2^ | < 4.2 × 10^-9^ |
| FSL B2-136 | | |  |  |  |  |  |  |
|  | 1 | | | 1.9 × 10^10^ | 0 (2 x 10^-2^) | NA^d^ | < 50 | < 2.6 × 10^-9^ |
|  | 2 | | | 2.8 × 10^10^ | 0 (1 x 10^-1^) | NA | < 10 | < 3.6 × 10^-10^ |
|  | 3 | | | 2.9 × 10^10^ | 0 (1 x 10^-1^) | NA | < 10 | < 3.5 × 10^-10^ |
|  |  | | |  |  |  |  |  |

**^a^**this column lists the actual number of putative kanamycin resistant colonies (i.e., colonies larger > 9 px) that were detected upon plating; the plating dilution used to determine colony counts is listed in parenthesis.

^b^this columns lists the number of kanamycin resistant revertants that occurred due to insertions or deletions in the *inlA* 5’polyA HT (as determined by DNA sequencing of the region of interest) and the number putative revertants (colonies) screened by sequencing

^c^The number of revertants per ml was calculated using the number of confirmed revertants from the previous column and the dilution factor; if only a proportion of putative revertants (colonies) was screened by sequencing the proportional fraction of true revertants was used to calculate this number.

^d^NA=not applicable; no colonies were tested for reversion since no putative revertants were obtained upon plating
